# Two Sides of the Same Coin: Distinct Sub-Bands in the α Rhythm Reflect Facilitation and Suppression Mechanisms during Auditory Anticipatory Attention

**DOI:** 10.1523/ENEURO.0141-18.2018

**Published:** 2018-09-17

**Authors:** Hesham A. ElShafei, Romain Bouet, Olivier Bertrand, Aurélie Bidet-Caulet

**Affiliations:** 1 Brain Dynamics and Cognition Team, Lyon Neuroscience Research Center; CRNL, Institut National de la Santé et de la Recherche Médicale Unité 1028, Centre National de la Recherche Scientifique Unité Mixte de Recherche 5292, Université de Lyon, Lyon, France 69500

**Keywords:** α, α sub-bands, attention, audition, magnetoencephalography, oscillations

## Abstract

Anticipatory attention results in enhanced response to task-relevant stimulus, and reduced processing of unattended input, suggesting the deployment of distinct facilitatory and suppressive mechanisms. α Oscillations are a suitable candidate for supporting these mechanisms. We aimed to examine the role of α oscillations, with a special focus on peak frequencies, in facilitatory and suppressive mechanisms during auditory anticipation, within the auditory and visual regions. Magnetoencephalographic (MEG) data were collected from fourteen healthy young human adults (eight female) performing an auditory task in which spatial attention to sounds was manipulated by visual cues, either informative or not of the target side. By incorporating uninformative cues, we could delineate facilitating and suppressive mechanisms. During anticipation of a visually-cued auditory target, we observed a decrease in α power around 9 Hz in the auditory cortices; and an increase around 13 Hz in the visual regions. Only this power increase in high α significantly correlated with behavior. Importantly, within the right auditory cortex, we showed a larger increase in high α power when attending an ipsilateral sound; and a stronger decrease in low α power when attending a contralateral sound. In summary, we found facilitatory and suppressive attentional mechanisms with distinct timing in task-relevant and task-irrelevant brain areas, differentially correlated to behavior and supported by distinct α sub-bands. We provide new insight into the role of the α peak-frequency by showing that anticipatory attention is supported by distinct facilitatory and suppressive mechanisms, mediated in different low and high sub-bands of the α rhythm, respectively.

## Significance Statement

We investigated the role of α oscillations, with a special focus on peak frequencies, in facilitatory and suppressive mechanisms during anticipation, using magnetoencephalographic (MEG) data collected during an auditory spatial attention task. We show, during anticipation of a visually-cued auditory target, a decrease in α power around 9 Hz in the auditory cortices, simultaneous to an increase around 13 Hz in in the visual regions, the latter significantly correlated with behavioral performances. Within the right auditory cortex, we show a larger increase in high α when attending an ipsilateral sound; and a stronger decrease in low α when attending a contralateral sound. Therefore, anticipatory attention would be supported by distinct facilitatory and suppressive mechanisms, mediated in different low and high α sub-bands, respectively.

## Introduction

We spend a large fraction of our time anticipating stimuli (Requin et al., 1991) and to support this behavior, anticipatory attention promotes the processing of upcoming relevant stimuli, resulting in reduced brain responses to unattended inputs and enhanced processing of relevant information (for review, see Hillyard et al., 1998). These modulations of target processing suggest the deployment of distinct facilitatory and suppressive attentional mechanisms during target expectancy, similarly to the inhibitory and facilitatory mechanisms supporting selective attention (de Fockert and Lavie, 2001; Gazzaley et al., 2005, 2008; Bidet-Caulet et al., 2007, 2010, 2015; Chait et al., 2010; Slagter et al., 2016). However, little is known about the potential facilitatory and suppressive attentional mechanisms activated during anticipation of an upcoming stimulus.

Oscillations in the α band, loosely defined between 8 and 14Hz, have been proposed to play a crucial role in anticipatory attention (for review, see Foxe and Snyder, 2011; Frey et al., 2015). Discovered in 1929 by Hans Berger (Berger, 1929), α oscillations were first considered a marker of cortical idling (Pfurtscheller et al., 1996). However, this idea has been challenged with α oscillations being assigned an active inhibitory role in cognitive processing (Klimesch et al., 2007; Jensen and Mazaheri, 2010). The large literature in the visual modality paints a rather dynamical picture in which, during target expectation, α power decreases in visual areas responsible for processing the attended space while α power increases in (1) visual areas responsible for processing the unattended space with or without distracting stimuli (Kelly et al., 2006; Rihs et al., 2007, 2009), and (2) areas responsible for processing unattended modalities (Foxe et al., 1998; Fu et al., 2001; Gomez-Ramirez et al., 2011; Jiang et al., 2015). Therefore, α oscillations would be a suitable candidate for supporting facilitatory and suppressive mechanisms of anticipatory attention.

Interestingly, distinct frequency peaks (sub-bands) in the α band manifest as a function of cortical location and task demand (Haegens et al., 2014). In a similar vein, Mazaheri et al. (2013) compared α activity while participants were cued to either a visual or an auditory target, and found a decrease in α power around 10 Hz in visual regions concomitant to an increase around 15 Hz, in the vicinity of the right auditory cortex. Taken together, these results highlight the importance of considering the frequency peak within the α band.

Contrary to the visual domain, only a handful of studies investigated the impact of anticipatory attention on α modulations in the auditory cortices. A recurrent magnetoencephalographic (MEG) finding is an increased α power, solely in the right auditory cortex, when attention was directed toward the ipsilateral right ear compared to when directed toward the contralateral ear (Müller and Weisz, 2012) or non-spatially oriented (Weisz et al., 2014). These results demonstrate how α oscillations could be involved in the suppressive mechanisms of auditory anticipatory attention (see also Frey et al., 2014; Weise et al., 2016), but do not shed much light on their implication in facilitatory mechanisms.

We aimed to examine the role of α oscillations in attentional facilitatory and suppressive mechanisms during auditory anticipation, within the auditory cortices and also between the visual and auditory regions. For this purpose, we recorded MEG activity during an auditory task in which spatial attention to auditory targets was manipulated by visual cues, either informative or not of the target side. By incorporating spatially uninformative cues, we aimed to delineate facilitating and suppressive mechanisms supporting auditory anticipatory attention (Bidet-Caulet et al., 2010).

We hypothesized that during a spatial attention task, the balance between facilitatory and suppressive mechanisms of auditory anticipatory attention would be indexed by α activity following two main patterns. (1) A decrease in α power (reflecting inhibition release, i.e., facilitation) in task-relevant auditory areas would be concomitant to an increase in α power (reflecting inhibition/suppression) in task-irrelevant visual cortices. (2) Within the right auditory cortex, we expected a decrease in α power when attention is directed toward the contralateral ear and an increase in α power when attention is directed toward the ipsilateral ear, relative to when attention is not spatially oriented (uninformative cues). Also, if distinct suppressive and facilitating attentional mechanisms are activated during anticipation, they should be differentially correlated to behavioral performances. Finally, to gain further insight into the role of the peak-frequency in the α band (Haegens et al., 2014), we aimed to systematically investigate the effect of the frequency peak with the prediction that facilitatory and suppressive attentional mechanisms would be mediated in different α sub-bands.

## Materials and Methods

### Participants

Fourteen healthy participants (eight females) took part in this study. The mean age was 25 years ± 0.85 SEM. All participants were right handed, and reported normal hearing, and normal or corrected-to-normal vision. All participants were free from any neurologic or psychiatric disorders. The study was approved by the local ethical committee, and subjects gave written informed consent, according to the Declaration of Helsinki, and they were paid for their participation.

### Stimuli and tasks

#### Competitive attention task (CAT)

**Figure 1.**
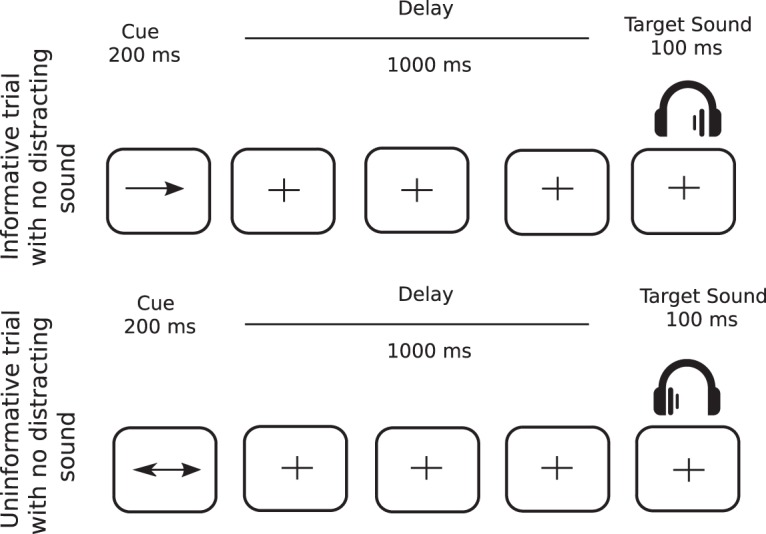
Protocol. Top row, In informative trials (67% of all trials), a one-sided visual cue (200-ms duration) indicated in which ear (left or right) the target sound will be played (100-ms duration) after a fixed 1000-ms delay. Bottom row, In uninformative trials (33% of all trials), a two-sided visual cue (200-ms duration) did not provide any indication in which ear (left or right) the target sound will be played. In 25% of all trials (not depicted in figure), a binaural distracting sound (300-ms duration), such as a phone ring, was played during the delay between cue and target.

In 75% of the trials, a target sound (100-ms duration) followed a central visual cue (200-ms duration) with a fixed delay of 1000 ms (Fig. 1). The cue was a green arrow, presented on a gray-background screen, pointing either to the left, right, or both sides. Target sounds were monaural pure tones (carrier frequency between 512 and 575 Hz; 5-ms rise time, 5-ms fall time). In the other 25%, the same structure was retained, however, a binaural distracting sound (300-ms duration) was played during the cue-target delay (50- to 650-ms range). However, for the purpose of this study, only distractor-free trials were analyzed. The cue and target categories were manipulated in the same proportion for trials with and without distracting sound. In 33.3% of the trials, the cue was pointing left and the target sound was played in the left ear, and in 33.3% of the trials, the cue was pointing right and the target sound was played in the right ear, leading to a total of 66.6% of informative trials. In the last 33.3% of the trials, the cue was uninformative, pointing in both directions, and the target sound was played in the left (16.7%) or right (16.7%) ear.

Participants were instructed to categorize two target sounds as either high- or low-pitched sound, by either pulling or pushing a joystick. The mapping between the targets (low or high) and the responses (pull or push) was counterbalanced across participants, but did not change across the blocks, for each participant. to account for the participants’ pitch-discrimination capacities, the pitch difference between the two target sounds was defined in a discrimination task (see below). Participants were informed that informative cues were 100% predictive and that a distracting sound could be sometimes played. They were asked to allocate their attention to the cued side in the case of informative cue, to ignore the distractors and to respond as quickly and correctly as possible. Participants had a 3.4-s (3400-ms) response window. In the absence of the visual cue, a blue fixation cross was presented at the center of the screen. Subjects were instructed to keep their eyes fixated on the cross and to minimize eye movements and blinks while performing the task.

#### Discrimination task

Participants were randomly presented with one of two target sounds: a low-pitched sound (512 Hz) and a high-pitched sound (575 Hz; two semitones higher), equiprobably in each ear (four trials per ear and per pitch). As described above, participants were asked to categorize the target sounds as either high- or low-pitched sound within 3 s.

#### Procedure

Participants were seated in a sound-attenuated, magnetically shielded recording room, at a 50-cm distance from the screen. The response device was an index-operated joystick that participants moved either toward them (when instructed to pull) or away from them (when instructed to push). All stimuli were delivered using Presentation software (Neurobehavioral Systems, RRID:SCR_002521). All sounds were presented through air-conducting tubes using Etymotic ER-3A foam earplugs (Etymotic Research, Inc.).

First, the auditory threshold was determined for the two target sounds differing by two semitones (512 and 575 Hz), in each ear, for each participant using the Bekesy tracking method (Von Békésy and Wever, 1960). The target sounds were then presented at 15-dB sensation level while the distracting sounds were played at 35-dB sensation level. Second, participants performed the discrimination task. If participants failed to respond correctly to >85% of the trials, the pitch of the high target sound was augmented, by half a semitone with a maximum difference of three semitones between the two targets (auditory thresholds were then measured with the new targets). Afterward, participants were trained with a short sequence of the CAT. Finally, MEG and EEG were recorded while subjects performed 15 blocks (72 trials each). Each trial lasted from 4.6–4.8 s, leading to a block duration of ∼5 min and a MEG/EEG session of ∼1 h 35 min (breaks included). After the MEG/EEG session, participants’ subjective reports regarding their strategies were collected.

### Behavioral data analysis

For behavioral data analysis, a response was considered correct, if it matched the response mapped to the target sound and was executed before the apparition of the following cue. The influence of the factor cue (three levels: left, right and uninformative) on the percentage of correct responses was tested using a linear mixed-effects models [lme4 package (Bates et al., 2014) for R Team, 2014; RRID:SCR_015654].

For *post hoc* analysis, we used the Lsmean package (Lsmean version 2.20-23; Searle et al., 1980) where *p* values were considered as significant at *p* < 0.05 and adjusted for the number of comparisons performed (Tukey method). Incorrect trials were excluded from further analysis, leaving on average (216 ± 6.92 SEM) trials per cue condition per participant. The influence of the cue on the median of reaction times (RTs) of the correct trials were tested using the same tests.

### Magnetoencephalography

#### Recordings

Simultaneous EEG and MEG data were recorded, although the EEG data will not be presented here. The MEG data were acquired with a 275-sensor axial gradiometer system (CTF Systems Inc.) with continuous sampling at a rate of 600 Hz, a 0- to 150-Hz filter bandwidth, and first-order spatial gradient noise cancellation. Moreover, eye-related movements were measured using vertical and horizontal EOG electrodes.

Head position relative to the gradiometer array was acquired continuously using coils positioned at three fiducial points; nasion, left and right pre-auricular points. Head position was checked at the beginning of each block to control head movements.

In addition to the MEG/EEG recordings, T1-weighted three-dimensional anatomic images were acquired for each participant using a 3T Siemens Magnetom whole-body scanner. These images were used for reconstruction of individual head shapes to create forward models for the source reconstruction procedures. The processing of these images was conducted using CTF’s software (CTF Systems Inc.).

#### Outline of the electrophysiological data analyses

The analyses reported here focused on modulations of oscillatory activity in the α band during top-down anticipatory attention, i.e., during the cue-target delay in trials with no distractor and a correct response. MEG data were pre-processed in the sensor space using the software package for electrophysiological analysis (ELAN Pack; Aguera et al., 2011). Further analyses were performed using Fieldtrip (www.fieldtriptoolbox.org; Oostenveld et al., 2011, RRID:SCR_004849), an open source toolbox for MATLAB (RRID:SCR_001622), custom-written functions and R (www.r-project.org; RRID:SCR_001905).

First, significant modulations of oscillatory activity in the α band after cue onset (cue-related activity) were assessed by contrasting post-cue activity against pre-cue activity in the sensor level time-frequency domain (see below, Sensor-level analysis).

Second, based on the sensor level results, two post-cue and one pre-cue time windows in two distinct frequency bands were chosen for source analyses (see below, Source-level analysis). Based on the results of post-/pre-cue contrast in the source domain, auditory and visual regions of interest (ROIs) were selected for further virtual electrode analysis, i.e., time-resolved estimation of source activity (see below, Defining ROIs and virtual electrodes). From these activities, we then specified the time courses, power spectrum, and the α peak frequency for each virtual electrode (see below, Reconstruction of source activity) and assessed the attentional modulations of the cue-related α activity (see below, Attentional modulation of α activity).

Third, correlation between RTs and cue-target activity was investigated in the sensor (see below, Correlation between α activity and behavioral data: sensor level) and source (see below, Correlation between α activity and behavioral data: source level) domains.

### Data pre-processing

#### Head movements

As participants had an EEG cap on, head movements were relatively more difficult to control, in comparison to standard MEG procedures, where the participant’s head is relatively stabilized to the MEG dewar via an inflatable cushion. Thus, in reference to the first block, head positions in the following blocks exceeded the pre-determined threshold of ±1 cm. This would have compelled us to exclude a huge portion of the trials if all 15 blocks were concatenated together. Therefore, for each subject, data were organized in three groups of five blocks so that, within each group, differences in head positions, recorded at the beginning of each block, did not exceed a threshold of ±1 cm.

It is noteworthy that for data pre-processing and sensor level analysis (described below) trials from the three groups were concatenated. However, for source level and virtual electrode analyses (described below), each group was processed separately, and outputs were eventually averaged.

#### Pre-processing

Only correct trials were considered for electrophysiological analyses. Data segments contaminated with muscular activity or sensor jumps were excluded semi-manually using a threshold of 2200 and 10,000 femtoTesla, respectively. Independent component analysis was applied on the bandpass filtered (0.1–40 Hz) data to remove eye-related (blink and saccades) and heart-related (ECG) artefacts. Subsequently, components (four on average) were removed from the non-filtered data via the inverse ICA transformation. Data were further notch filtered at 50, 100, and 150 Hz and high-pass filtered at 0.1 Hz.

### Cue- and target-related activity

#### Sensor-level analysis

To investigate the dynamics of α power modulations after the visual cue, the oscillatory power of trials from the three cue conditions all together was calculated using Morlet Wavelet decomposition with a width of four cycles per wavelet (m = 7; Tallon-baudry and Bertrand, 1999) at center frequencies between 5 and 18 Hz, in steps of 1 Hz and 50 ms. Activity of interest (defined between 0 and 2 s post-cue and 7–15 Hz) was contrasted against mean baseline activity (−0.6 to −0.2 s pre-cue) using a nonparametric cluster-based permutation analysis (Maris and Oostenveld, 2007). In brief, this test first calculates paired *t* tests for each sensor at each time-frequency point, which are then thresholded at *p* < 0.05. The sum within each cluster (Tsum) is retained, and the procedure is repeated 1000 times on shuffled data in which the condition assignment within each individual swapped randomly. On each permutation, the maximum Tsum (Tmax) is retained yielding a distribution of 1000 Tmax values. From this distribution, the cluster probability of each empirically observed Tsum can be derived. Clusters are labeled as significant with *p* ≤ 0.05. Please note, that for this test, cluster permutations control for multiple comparisons in time, frequency and sensor space dimensions.

#### Source-level analysis

To elucidate the possible brain regions underlying the sensor-level α modulations, we have defined two post-cue (0.2–0.6, 0.6–1.0) and one pre-cue (−0.6 to −0.2) time-windows in two different frequency bands (9 and 13 ± 2 Hz). These time-frequency windows have been chosen based on the results from the statistical contrast in the sensor level.

To estimate the brain regions driving activity in these time-frequency windows, we have used the frequency–domain adaptive spatial technique of dynamical imaging of coherent sources (DICS; Gross et al., 2001). Data, from all conditions, within each group of blocks were concatenated, and cross-spectral density (CSD) matrix (−0.7 to 2 s, relative to cue onset) were calculated using the multitaper method with a target frequency of 11 (±4) Hz.

For each participant, an anatomically realistic single-shell headmodel based on the cortical surface was generated from individual head shapes (Nolte, 2003). A grid with 0.5-cm resolution was normalized on a MNI template, and then morphed into the brain volume of each participant. Leadfields for all grid points along with the CSD matrix were used to compute a common spatial filter that was used to estimate the spatial distribution of power for all time-frequency windows of interest per group of blocks. For each participant, these power distributions were averaged across the three groups of blocks. Afterward, Each post-cue window was contrasted against a corresponding baseline pre-cue window using a nonparametric cluster-based permutation analysis (Maris and Oostenveld, 2007). For this test, cluster permutations control for multiple comparisons in the source space dimension.

#### Defining ROIs and virtual electrodes

The aforementioned source-level analysis provides a snapshot picture of underlying cortical activity. To go a step further, we defined virtual electrodes within ROIs, for the purpose of resolving the time course of activity at the source level. The source space was subdivided into 116 anatomically defined ROIs according to the macroscopic anatomic parcellation of the MNI template using the automated anatomic labeling (AAL) map (Tzourio-Mazoyer et al., 2002). We limited our analysis to four auditory regions; left and right Heschl gyri (HG) and superior temporal gyri (STG) and two occipital regions (left and right middle/superior gyri). For each auditory region; virtual electrodes were defined as the average of five neighboring voxels exhibiting the strongest α power modulations, i.e., highest *t* values in the source-level baseline contrast in the 0.6 to 1s (relative to cue onset) and 7- to 11-Hz time-frequency window. Same procedure was used for the occipital regions; however, voxels were chosen based on the highest *t* values in the source-level baseline contrast in the 0.6- to 1-s (relative to cue onset) and 11- to 15-Hz time-frequency window.

#### Reconstruction of source activity

To get a time-resolved estimation of source activity, we computed the time-frequency signal at the virtual electrode (defined above) level using the LCMV beamformer. Spatial filters were constructed from the covariance matrix of the averaged single trials at sensor level (−0.7 to 2 s, relative to cue onset, 1–20 Hz, λ 15%) and the respective leadfield by a linearly constrained minimum variance (LCMV) beamformer (Van Veen et al., 1997). Afterward, spatial filters were multiplied by the sensor level data to obtain the time course activity at each voxel of interest. Activity was averaged across the five voxels defined for each ROI (see section above) and for each hemisphere. Moreover, activity was averaged across the two auditory ROIs (HG and STG). Thus, limiting our analysis to four ROIs (one auditory and one occipital in each hemisphere).

For each ROI, we subtracted the evoked potential (i.e., the signal averaged across all trials) from each trial. Subsequently, time-frequency power was calculated in the same manner as in the sensor level analysis using Morlet Wavelet decomposition.

To visualize the different profiles observed on both sensor and source levels, α power (computed using Morlet Wavelets) was averaged between 7 and 11 Hz, and between 11 and 15 Hz, for auditory and visual regions, separately, to extract the time course of α activity in these two α sub-bands.

In addition, α power (computed using Morlet Wavelets) was averaged between 0.6 and 1s for each ROI, to extract the power spectrum in each subject. Afterward, individual α peak frequency (iAPF) was defined separately for auditory and visual regions, in each subject. For auditory virtual electrodes, the peak was defined as the frequency with the maximum α power decrease relative to the baseline (−0.6 to −0.2 s pre-cue onset) between 5 and 15 Hz. For visual virtual electrodes, the peak was defined as the frequency with the maximum α power increase relative to the baseline. The median APFs across subjects and hemispheres were 9 and 13 Hz in the auditory and visual virtual electrodes, respectively.

#### Attentional modulation of α activity

A linear mixed-effects model (lme) was fit to predict modulation of α activity uniquely in auditory virtual electrodes between 600 and 1000 ms (relative to cue onset) with the following factors as fixed effect: (1) cue laterality according to the auditory cortices (three levels: ipsilateral, contralateral, and uninformative); (2) hemisphere (two levels: left and right); and (3) frequency (two levels: 9 and 13 Hz). A random effect was included for each participant and thus allowing us to model variability between participants. The chosen frequencies were the median APFs calculated in the previous analysis (see above, Reconstruction of source activity). Similar to the previous step, for *post hoc* analysis, we used the Lsmean package.

#### Correlation between α activity and behavioral data: sensor level

As a final step, and to assess the relationship between the cue-related changes in α power, in the sensor space, and RTs, correlation topographies were created (Mazaheri et al., 2013). First, we performed a trial-by-trial correlation, using non-parametric Spearman tests, in each participant, between RTs and post-cue α power at each time frequency point (between 6 and 16 Hz, by steps of 1 Hz, and between 0 and 1200 ms post-cue onset, by steps of 50 ms) for each sensor, to create topographies of the correlation (Mazaheri et al., 2013). The correlation coefficients were subsequently converted to *z* values using Fisher’s r- to z-transformation to obtain a normally distributed variable. The statistical significance of the correlations was assessed at the group level with a one-sample *t* test of the correlation *z* values at each sensor and each time-frequency point, and then subjected to a cluster-level randomization test to correct for multiple comparisons in the sensor space, time, and frequency dimensions.

#### Correlation between α activity and behavioral data: source level

To assess the relationship between cue related changes in α power and RTs in source-space, single trial α activity was reconstructed at each grid point using a partial cannonical correlation (PCC) beamformer, a more computationally efficient alternative to the DICS beamformer. Afterward, we performed a trial-by-trial correlation, using non-parametric Spearman tests, in each participant, between RTs and post-cue α power (between 10 and 16 Hz, and between 900 and 1200 ms, according to the sensor level results) at each grid point (Mazaheri et al., 2013). The correlation coefficients were subsequently converted to *z* values using Fisher’s r- to z-transformation to obtain a normally distributed variable. The statistical significance of the correlations was assessed at the group level with a one-sample *t* test of the correlation *z* values at each grid point and then subjected to a cluster-level randomization test to correct for multiple comparisons in the source space dimension.

### Power analysis

To demonstrate the statistical robustness of our tests (see above, Behavioral data analysis and Attentional modulation of α activity), we have applied sensitivity power analyses using the G*Power software (Faul et al., 2007, 2009), using a power of 0.8, an α error of 0.05, and correlation of 0.5 among repeated-measures; for all the analysis based on linear mixed-effects models (as an approximation), we ran the sensitivity power analysis for a repeated-measures ANOVA. Results are detailed in relevant sections.

## Results

### Behavioral analysis

**Figure 2.**
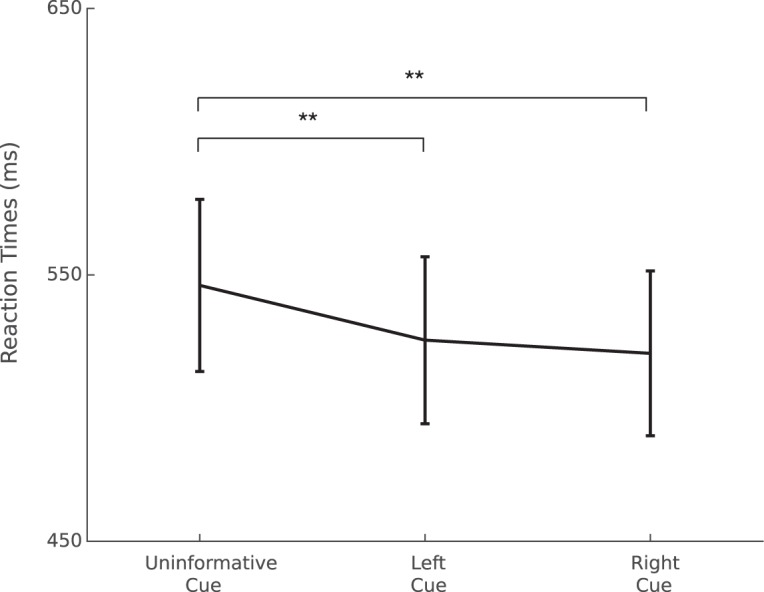
Mean of median RT (ms) per condition; **p < 0.01. Error bars represent SEM.

The percentage of correct responses (on average: 96.05 ± 0.73 SEM) was not significantly modulated by the cue category. For the median RTs, as shown in Figure 2, we found a significant main cue effect (*F*_(2,26)_ = 31.5, *p* < 0.01, η^2^ = 0.7). The reported effect size (f; Cohen, 1988) of this test is 1.52 superior to the required effect size of 0.35 as calculated by the G*Power software.

*Post hoc* tests indicated that participants were faster when the cue was informative (either right or left) in comparison to the uninformative cue (*p* < 0.01). No significant differences were found between the left and right cue conditions.

### Cue- and target-related α activity: sensor level analysis

**Figure 3.**
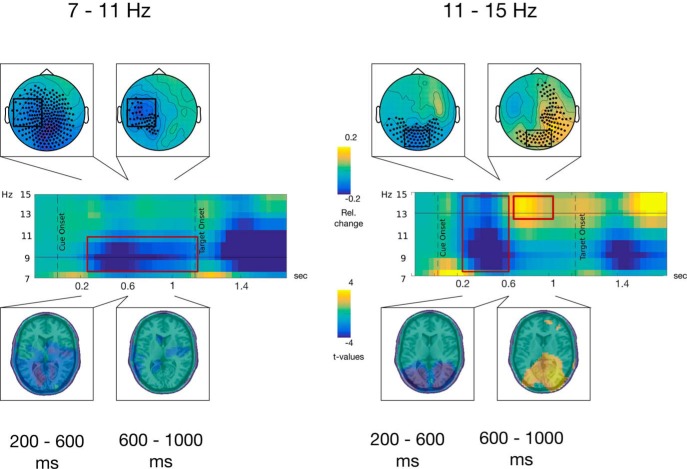
Comparison between low (7–11 Hz; left panel) and high (11–15 Hz; right panel) α activity. First row, Topographical maps of baseline corrected (−600 to −200 ms pre-cue onset) α power averaged in the respective frequency bands during two latency windows: (1) 200–600 ms and (2) 600–1000 ms, relative to cue onset. Sensors highlighted with black dots present α activities statistically significant from the baseline using cluster-based permutation tests and sensors highlighted by black boxes were used to represent the time-frequency activity in the second row. Second row, Time-frequency representations of α power baseline corrected (−600 to −200 ms pre-cue onset) averaged across sensors highlighted by the black boxes over the topographical maps on the first row. Third row, Distributions of *t* values, masked at *p* < 0.05, from cluster-based permutation tests contrasting time-frequency windows of interest against baseline activity at the source level.

On contrasting post-cue activity to baseline activity, two profiles centered on two distinct frequencies (9 and 13 Hz; low and high α) were distinguished. In the low α frequencies, a widespread decrease lasted between cue onset and 600 ms (post-cue-onset; early period). Later on, this activity was spatially focused to left temporo-parietal sensors just before the target onset (late period). Simultaneously, in the high α frequencies, the early period displayed an occipitally focalized decrease followed by an increase that spreads to right temporal sensors in the late period (Fig. 3).

### Cue- and target-related α activity: source level analysis

Sources of these activities were estimated and contrasted to the baseline window. In the early period (200–600 ms), a general decrease of the low-α can be observed in several occipital, temporal and central brain regions, bilaterally (Table 1). However, in the same time period, at higher α frequency, this decrease was restricted to regions dedicated to visual processing in the occipital and temporal lobes (Table 1). In the late period, the low-α decrease became more restricted to the auditory regions in the temporal cortices, e.g., bilateral HG and STG, and to motor areas (Table 1). However, at higher frequencies, an α increase was maximal in occipital, parietal and temporal regions dedicated to visual processing, and in parietal regions (Table 1).

**Table 1.** Brain regions displaying significant α activity modulations in the low (7–11 Hz) or high (11–15 Hz) α frequency bands in two time windows on baseline contrast in the source level

7–11 Hz	Early time window (200–600 ms)	Late time window (600–1000 ms)
	Left and right ↓: Heschl gyrus Inferior, middle, and STG calcarine Cuneus Inferior, middle, and superior occipital gyri Inferior parietal gyrus Postcentral gyrus Precentral gyrus Precuneus Supp. motor area	Left and right ↓: Heschl gyrus Inferior, middle, and STG Inferior parietal gyrus Postcentral gyrus Precentral gyrus Supp. motor area
11–15 Hz	Early time window (200–600 ms)	Late time window (600–1000 ms)
	Left and right ↓: Calcarine Cuneus Inferior and middle occipital gyri Inferior and middle temporal gyri	Left and right ↑: Calcarine Cuneus Precuneus Inferior and middle occipital gyri Inferior and middle temporal gyri Inferior and superior parietal gyri

Up-arrows indicate α synchronization (relative increase in power) while down-arrows indicate α desynchronization (relative decrease in power).

### ROI analysis: α time course and peak frequency

**Figure 4.**
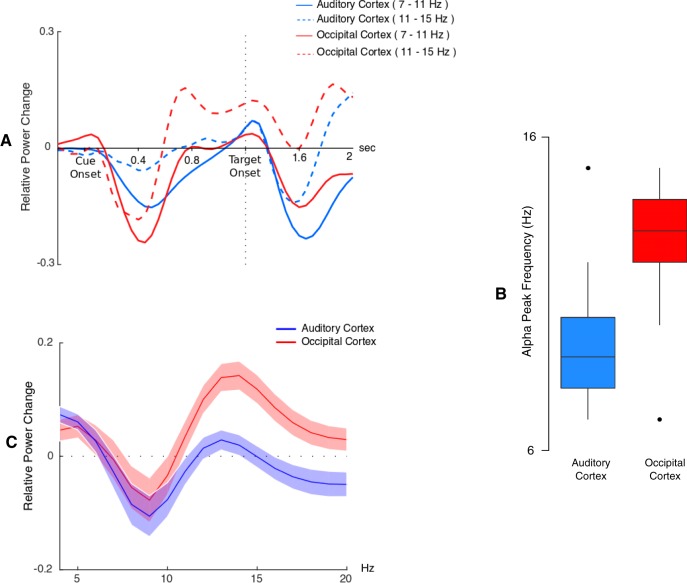
Source level activity. ***A***, Time course of α (relative to baseline) between 7–11 and 11–15 Hz, for occipital and auditory virtual electrodes averaged across both hemispheres. Note that subtracting the evoked response from single trials before time-frequency transformation only partially removed the evoked response to the target in the α bands. ***B***, Boxplot of Individual α peak frequency in visual and auditory regions. ***C***, Relative power spectrum averaged between 600 and 1000 ms post-cue in visual and auditory regions.

At the virtual electrode level, we were able to confirm that the time-frequency profiles of both auditory and visual ROIs are consistent with the profiles that have been demonstrated at the sensor level (Fig. 4*A*). We could also confirm, at the virtual electrode level, the frequency differences that have been observed at the sensor level. Indeed, the median α frequency peak across subject was 9 Hz in auditory cortices and 13 Hz in visual cortices (Fig. 4*B*). Moreover, as can be observed in Figure 4*C*, these α peak frequencies were well circumscribed within the 7- to 15-Hz α band.

### ROI analysis: attentional modulations of α activity

In order investigate the modulation of α activity in auditory virtual electrodes, a lme model was used with three factors: (1) cue laterality according to the auditory cortices (three levels: ipsilateral, contralateral and uninformative); (2) hemisphere (two levels: left and right); and (3) frequency (two levels: 9 and 13 Hz).

The lme model yielded several significant main effects and interactions (listed in Table 2 with interaction of interest in bold font). The highest-order significant interaction of interest is the three-level interaction between cue laterality, hemisphere, and frequency (*F*_(2,26)_ = 3.07, *p* = 0.04, η^2^ = 0.17). The reported effect size (f) of this test is 0.65 superior to the required effect size of 0.28 as calculated by the G*Power software.

**Table 2.** Significant results of the LME model testing the modulation of α activity by cue laterality, hemisphere, and frequency

Factor	*p* value	*f* statistic
Hemisphere	0.02	5.3
Frequency	<0.001	141
Cue laterality by hemisphere	0.01	4.0
**Cue laterality by hemisphere by frequency**	**0.04**	**3.1**

The interaction of interest is highlighted in bold.

**Figure 5.**
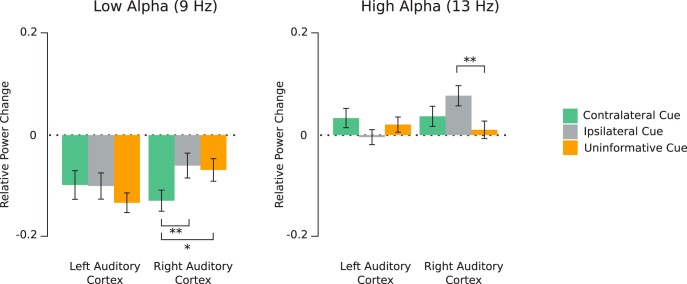
α Power (relative to baseline) averaged between 600 and 1000 ms (post-cue onset) at 9 Hz (left panel) and 13 Hz (right panel) for the three cue conditions; *p < 0.05, **p < 0.01. Error bars represent SEM.

To elucidate this interaction, we performed *post hoc* lme models testing the influence of the cue laterality (three levels: ipsilateral, contralateral, and uninformative) and hemisphere (two levels: left and right), for each frequency (9 and 13 Hz), since we aimed to shed more light onto the role of peak frequencies on α modulations (Fig. 5)

At 9 Hz (low α), only the two-level interaction between cue laterality and hemisphere (*F*_(2,26)_ = 5.2, *p* = 0.005, η^2^ = 0.17) reached significance. The reported effect size (f) of this test is 0.45 while the required effect size as calculated by the G*Power software was 0.23; 2 by 2 *post hoc* testing revealed that in the right hemisphere (auditory cortex), α power was significantly lower in the contralateral cue condition, in comparison to the ipsilateral and uninformative cue condition (*p* = 0.004 and *p* = 0.01, respectively). No significant effects were found in the left hemisphere (Fig. 5). In summary, a facilitatory effect on the low α power was found in the right auditory cortex for the contralateral cue.

At 13 Hz (high α), only the two-level interaction between cue laterality and hemisphere (*F*_(2,26)_ = 4.95, *p* = 0.007, η^2^ = 0.1) reached significance. The reported effect size (f) of this test is 0.33 while the required effect size as calculated by the G*Power software was 0.28; 2 by 2 *post hoc* testing revealed that in the right hemisphere (auditory cortex), α power was significantly higher in the ipsilateral cue condition, in comparison to the uninformative cue condition (*p* = 0.007), but not to the contralateral cue condition (*p* = 0.16). No significant effects were found in the left hemisphere (Fig. 5).

In summary, a suppressive effect on the high α power was found in the right auditory cortex for the ipsilateral cue.

### Correlation between α activity and behavioral data

**Figure 6.**
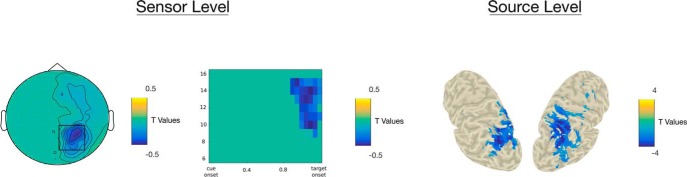
***A***, Topography of *t* values, masked at *p* < 0.05, from cluster-based permutation testing of the significance of the correlation between α activity (900–1200 ms, 10–15 Hz) and RT at the sensor level. ***B***, Time-frequency distribution of *t* values, masked at *p* < 0.05, averaged across sensors highlighted by the black box on the topography in ***A***. ***C***, *t* values source distributions, masked at *p* < 0.05, from cluster-based permutation testing of the significance of the correlation between α activity (900–1200 ms, 10–16 Hz) and RT. Please note, that negative *t* values signify negative correlation between RTs and α activity.

At the sensor level, pre-target activity between 0.9 and 1.2 s (relative to cue onset) in the 10- to 15-Hz frequency band at a cluster centered around right occipital and parietal sensors was found to negatively correlate with RTs (*p* = 0.001). In other words, the higher individual α power in that cluster, the faster the participant. At the source level, α activity between 0.9 and 1.2 s (relative to cue onset) and 10–16 Hz, mainly in the left and right superior occipital gyri, the left middle occipital gyrus, the right calcarine, and the right postcentral gyrus, was found to negatively correlate with RTs (*p* = 0.01; Fig. 6).

## Discussion

In this study, we have demonstrated that (1) anticipating a visually-cued auditory target differentially modulates α power in the auditory and visual cortices; (2) these modulations occur within different α sub-bands; (3) modulations in the right auditory cortex (facilitation and suppression) also occur within different α sub-bands; and (4) RTs to the auditory target correlate with the α power increase in the visual cortices.

### Behavioral measure of top-down anticipatory attention

Participants identified the target pitch faster in trials with an informative cue, in agreement with several previous studies (Golob et al., 2002; Bidet-Caulet et al., 2015). This effect is more likely to be related to differences in anticipatory attention since the informative cue provided additional information solely about the location of the target and not about its category neither its mapped response, thus motor preparation was equivalent across all conditions.

### Distinct profiles of α activity in visual and auditory regions

In line with our hypothesis, anticipating an auditory target modulated α power differently in the auditory and visual cortices, following different patterns. In the auditory cortex, after the visual cue onset, low-frequency α (∼9 Hz) power continuously decreased until target onset. Simultaneously, in the visual cortices, a transient decrease in low and high-frequency (∼13 Hz) α power between 200 and 600 ms post-cue onset was followed by a power increase, uniquely in high α, before target onset.

According to recent hypotheses, α oscillations reflect regulation of cortical excitability (Klimesch et al., 2007; Jensen and Mazaheri, 2010; Foxe and Snyder, 2011). This gauge would be supported by α power increases in task-irrelevant regions and by α power decreases in task relevant regions. In line with previous findings in the visual (Sauseng et al., 2005; Thut, 2006), somatosensory (Haegens et al., 2012), auditory (Müller and Weisz, 2012; Weisz et al., 2014), and audiovisual (Mazaheri et al., 2013; Frey et al., 2014; Van Diepen and Mazaheri, 2017) domains, we have found that anticipating an auditory target resulted in (1) a decrease in α power, possibly leading to increased excitability, in task-relevant auditory cortical regions, simultaneous to (2) an increase in α power, probably reducing excitability, in task-irrelevant visual regions.

### Top-down modulation of α activity in the auditory cortex

A scant literature (Gomez-Ramirez et al., 2011; Weisz et al., 2011, 2014; Müller and Weisz, 2012; Frey et al., 2014; Weise et al., 2016), mostly using MEG, exists on α generators in the auditory cortices, probably due to the limitations of EEG technique to capture their activity (Frey et al., 2014). In the present study, using MEG, we could show not only that α activity, in the auditory cortices, is modulated according to the visual cue information, but also that these modulations occur within different α sub-bands.

In the auditory cortices, to optimize the processing of an upcoming monaural sound, two phenomena might be expected: (1) an inhibition (increase in α activity) in the auditory cortex ipsilateral to the attended side, and (2) a pre-activation (or released inhibition, i.e., decrease in α power) in the contralateral auditory cortex. The question is: which of these two modulations (down- or upregulation) would drive anticipatory attention? By incorporating an uninformative cue condition, we could delineate these facilitatory and suppressive mechanisms.

We observed α power modulations according to the visual cue information, in the right auditory cortex, only. At lower α frequencies (∼9 Hz), we found a decrease in α power (relative to the baseline), in the three cue conditions (contralateral, ipsilateral and uninformative). Importantly, this decrease was most prominent when a contralateral sound was expected rather than an ipsilateral or a spatially non-cued sound. On the other hand, at higher α frequencies (∼13 Hz), an increase in α power (relative to the baseline) was observed in all conditions. Interestingly, this increase was more prominent when an ipsilateral, rather than a spatially non-cued target was expected.

The present results corroborate previous findings (Müller and Weisz, 2012; Weisz et al., 2014) showing that the right auditory cortex plays a special role in auditory spatial attention. We extend these findings by demonstrating that the excitability of the right auditory cortex can be both (1) downregulated for processing an ipsilateral right-ear sound and (2) upregulated for processing a contralateral left-ear sound. Importantly, to our knowledge, the present study is the first one to demonstrate that these modulations occur at different α frequencies, suggesting that the dynamic equilibrium between suppressive and facilitatory mechanisms of auditory anticipatory attention would be supported by different high and low α sub-bands, respectively.

Finally, α activity in the left auditory cortex was not modulated by top-down attention. This asymmetry could be interpreted in the light of the right hemispheric specialization in pitch processing (Milner, 1962; Zatorre and Belin, 2001; Zatorre et al., 2002; Lattner et al., 2005; Hyde et al., 2008). Since participants performed a pitch categorization task, the right auditory cortex would be more relevant for target sound processing and thus more influenced by top-down attention. The asymmetry of α activity modulations could also be interpreted in the light of the right hemispheric dominance in spatial attention that has been illustrated for the auditory (Zatorre and Penhune, 2001; Spierer et al., 2009) and visual (Nobre et al., 1997; Corbetta and Shulman, 2002) modalities. This dominance would reflect a functional asymmetry in auditory processing, wherein the left auditory cortex preferentially processes sounds within the contralateral egocentric space, whereas the right auditory cortex processes the entire acoustic space (Spierer et al., 2011).

### Correlation between α activity and behavioral performances

We found that the higher α power in the occipital cortices, the faster participants correctly discriminated the upcoming target sound. In other words, the stronger inhibition of task-irrelevant regions, the faster the subjects. This result is in line with previous findings that behavioral performances correlate with the increase in α power (Haegens et al., 2012) and reinforces the hypothesis that α oscillations exert an inhibitory role (Jensen and Mazaheri, 2010; Klimesch, 2012). Importantly, this correlation between an increase in α power in irrelevant brain regions and behavior was only found significant in the higher α frequencies (10–15 Hz), bringing further evidence for a specificity of the high α sub-band in suppressive attentional mechanisms.

Contradictory to the present findings, a positive correlation between α power in the auditory cortices and RTs in a sound discrimination task was found in a previous study (Mazaheri et al., 2013). However, differences between the two studies might explain this discrepancy. First, in their study, spatial attention was not modulated, i.e., the auditory target was always binaural. Second, participants discriminated three auditory target frequencies that were further apart in pitch and much easier to discriminate in comparison to our paradigm (250, 1000, and 4000 Hz vs 512 and 575 Hz). We posit that in the case of an easy task the excitability of relevant areas can be up and down regulated and correlate with task performance; whereas in the case of a difficult task, the excitability of relevant areas would be maximal and only the inhibition of signal dispersion to irrelevant areas could fluctuate and correlate with performance.

### The role of different α frequency sub-bands

The present study highlights specificities of low and high α sub-bands: (1) the peak frequency of the α increase in visual regions was found to be higher (∼13 Hz) in comparison to that of the α decrease in auditory regions (∼9 Hz); (2) the α increase in visual regions was found to be significantly correlated to behavior in the high α frequencies, only; (3) in the right auditory cortex, a larger decrease in α power during contralateral sound expectation was found in the low α, whereas a stronger increase in α power during ipsilateral sound anticipation was found in the high α. The existence of different sub-bands of the α rhythm is not a new concept (Klimesch et al., 1993, 1999; Sauseng et al., 2005; Groppe et al., 2013), but their functional role is still unclear. Recently, α generators have been observed in each of the cortical laminae (Haegens et al., 2015) in primary sensory cortices. Interestingly, the α activity seems to peak at different frequencies according to the layers, providing neuronal underpinnings to different α sub-bands. In the present study, the differences observed across frequencies can be interpreted differently by considering the α peak frequency as a “trait” or “state” variable (Haegens et al., 2014), providing information into their functional role, as discussed in the following.

The present results of different dominant frequencies in the visual and auditory regions are in line with evidence from previous studies demonstrating that α peak frequency varies as a function of cortical location (Kawasaki et al., 2010; Haegens et al., 2014). α Peak frequency could be considered as a trait or a “characteristic” variable that changes across individuals (Klimesch, 1999; Başar, 2012) and cortical regions, as found during resting state, in parietal and occipital regions (Haegens et al., 2014). In this light, the differences in α peak frequency reported here might be related to anatomic and physiologic disparities between the visual and auditory cortices. However, one should note that no difference in α peak frequencies was found between the macaque auditory, visual and somatosensory primary areas (Haegens et al., 2015).

Nonetheless, the present findings also show an increase in high α power, when attending an ipsilateral sound, in the right auditory cortex. This is in agreement with the results of Mazaheri et al. (2013) pointing to an α activity increase in the vicinity of the auditory cortices to be centered around higher α frequencies. Therefore, α peak frequency could also be considered as a state variable that would index performance fluctuations, cognitive demands and probably the functional task-relevance of a certain cortical region (Klimesch, 1999; Başar, 2012; Haegens et al., 2014). The present results show that suppressive attentional mechanism in the visual non-relevant regions are indexed by an increase in high α power which is correlated to behavior. Moreover, within the right auditory cortex, suppression (downregulation) of brain activity when attending an ipsilateral sound is reflected in the high α sub-band; whereas brain processing facilitation of the contralateral expected sound is indexed in the low α sub-band. Taken together, the present results highly suggest that different high and low α sub-bands would support suppressive and facilitatory mechanisms of anticipatory attention, respectively.

## Conclusion

The current study replicates and extends previous findings of the presence of α generators in the auditory cortices and of the right hemispheric dominance of auditory spatial attentional modulations.

Importantly, the present work provides evidence of distinct facilitatory and suppressive mechanisms supporting anticipatory attention. These two attentional mechanisms have distinct timing in task-relevant and task-irrelevant brain areas, are differentially correlated to behavior, and are supported by different sub-bands of the α rhythm.

Therefore, the present findings provide new insight into the role of the peak-frequency in the α band by showing that anticipatory attention is a dynamic process supported by a balance between facilitatory and suppressive mechanisms, which would be mediated in different low and high sub-bands of the α rhythm, respectively.

## Synthesis

Reviewing Editor: Morgan Barense, University of Toronto Decisions are customarily a result of the Reviewing Editor and the peer reviewers coming together and discussing their recommendations until a consensus is reached. When revisions are invited, a fact-based synthesis statement explaining their decision and outlining what is needed to prepare a revision will be listed below. The following reviewer(s) agreed to reveal their identity: Aneta Kielar.

We all agreed that the manuscript has the potential to make an important contribution to the field that will be of broad interest. However, we also agreed that there are important concerns that must be addressed with additional analyses and revisions to improve clarity. The reviewers' specific comments are pasted below.

Reviewer 1:

In this paper authors investigated the modulation of high and low bands of alpha activity in response to the anticipatory cue. The results indicated that alpha power at these two alpha frequency bands was responsive to the presentation of the attentional cue. The responses differed depending on the time window and were regionally specific. In the 200-600 ms after the cue presentation, there was a widespread alpha power suppression in the 7-11 Hz range, whereas the 11-15 Hz frequencies were decreased in the occipital regions. In the latter time window (600-1000 ms), the responses differentiated, such that the low alpha was suppressed in the auditory regions and high alpha was enhanced in the visual regions. These effects correlated with reaction times. The results indicate that modulation of alpha reflects facilitatory and suppressive mechanism during stimulus anticipation the regionally specific manner.

This manuscript contributes to a better understanding of the role of alpha oscillations in the modulation of cortical function. The data illustrate facilitatory and suppressive mechanism of alpha band oscillations.

I noted a few areas that may need clarification.

Design:

What was the purpose of condition with the distracting sound? If uninformative cue was always presented with the distracting sound this may have had an effect on responses. What were the effects of right vs. left ear presentation for the uninformative cue condition?

Analyses/results section

I think that the paper could be improved by organizing data analysis section better. The different sections seem somewhat disconnected from each other. Maybe having a sentence at the start of each subsection explaining the purpose of each analysis described would help.

Under section 2.6.4 it is stated that alpha power was analyzed in the two frequency bands 7-11 Hz and 11-15Hz. Why did authors chose to overlap the two bands?

It would be helpful to the reader if Table 1 indicated increases and decreases at different the frequency bands.

In Figure 6 alpha was averaged in different time window 900-1200 ms and from 10-12 Hz. It was not completely clear from the text why time window and frequency range changed?

I wonder if authors could use a better brain model in Figure 6C to overlay the results of source analysis.

The correlational analyses are interesting. However, I could not tell how/whether they were corrected for the multiple comparison.

Reviewer 2:

This is a carefully collected study providing a nice replication of a reported finding on alpha power fluctuations regulating activity beyond the visual modality. As such strengthening the notion of alpha activity organizing functional brain architecture. The reported findings are of relevance for a broad readership and will strengthen understanding and further theoretical development in the field of cognitive neuroscience. However, several points need to be addressed:

1. “Only this power increase in high alpha was found correlated with behavior” ;. This specificity claim required test of the homogeneity of regression slopes. This test is lacking yet crucial.

2. Saccades are not controled. It is now well established that saccades can trigger and affect alpha power fluctuations substantially. Please calculate and provide an illustration of saccade direction as a function of left, right, and uninformative cue. This can be done either on the EOG or frontal SQUIDS data.

3.Which hand was used for joystick control? It is well established that executiion and/or preparation for motor output is associated with contra lateral decrease in high alpha / beta power in parallel to ipsi lateral increase. This is somewhat the pattern of activity illustrated in Figure 6A for example.

4. Averaging of time courses within ROIs is dangerous due to different (arbitrary) polarities of beamforming results. I suggest TFR prior to averaging across dipoles within ROIs. Does this affect Figure 5? Also size asymmetry between left < right Heschl's gyrus would likely bias power estimates. Yet, these are the basis for the right AC claim. How many dipoles were included per auditory parcel? Was that done for Heschl Gurys or also Planum temporale included?

5. What was the regularization parameter for DICS? Please re-compute with smallest value possible or even without any regularization on the raw non-ICAed data. Are there “real” activations of AC or a “leakage” of posterior activation due to regularization?

6. Correlation between alpha and reaction time is computed using sliding window of 100 ms. This gives a frequency resolution of 0 10 20 30 etc. Hz. Yet 7 and 15 are not on that list. Please re-calculate this analysis using fft and not sliding window approach

over the entire 1.2 sec period. This gives the desired freq resolution in steps of 1 Hz and allows for reliably estimated power values.

7. Correlations with behavior in figure 6 flip polarity from sensor to source space- why?

8. Please explain what “Partial Cannonical Correlation (PCC) beamformer” is and how it works. Also why using PCC and not LCMV or DICS filters multiplied with single trial sensor data?

## Author Response

We thank the reviewers for their constructive comments on our manuscript entitled “Two sides of the same coin: Distinct sub-bands in the alpha rhythm reflect facilitation and suppression mechanisms during auditory anticipatory attention” ;. Please find below the reviewers' comments (in blue font) along with our replies in black. In the revised manuscript, modifications are highlighted in yellow.

Reviewer #1: In this paper authors investigated the modulation of high and low bands of alpha activity in response to the anticipatory cue. The results indicated that alpha power at these two alpha frequency bands was responsive to the presentation of the attentional cue. The responses differed depending on the time window and were regionally specific. In the 200-600 ms after the cue presentation, there was a widespread alpha power suppression in the 7-11 Hz range, whereas the 11-15 Hz frequencies were decreased in the occipital regions. In the latter time window (600-1000 ms), the responses differentiated, such that the low alpha was suppressed in the auditory regions and high alpha was enhanced in the visual regions. These effects correlated with reaction times. The results indicate that modulation of alpha reflects facilitatory and suppressive mechanism during stimulus anticipation the regionally specific manner. This manuscript contributes to a better understanding of the role of alpha oscillations in the modulation of cortical function. The data illustrate facilitatory and suppressive mechanism of alpha band oscillations. I noted a few areas that may need clarification.

What was the purpose of condition with the distracting sound? If uninformative cue was always presented with the distracting sound this may have had an effect on responses. What were the effects of right vs. left ear presentation for the uninformative cue condition?

The paradigm used, The Competitive Attention Task, has been designed to assess both bottom-up and top-down mechanisms of auditory attention and the interaction between these mechanisms. Hence, the presence of a distracting sound in only 25% of trials. However, these distracting sounds were played equiprobably across all cue conditions (left, right and uninformative). In other words, while in Figure1, only two types of trials were presented, distracting sounds were played only in 25% of the trials with an uninformative cue.

For the purpose of this study, to investigate the facilitatory and suppressive mechanisms of top-down auditory attention, we have focused only on distractor-free trials. Therefore, in order to clear up any future confusion, we have modified the figure 1 to depict only distractor-free trials (see below).

In the uninformative cue condition, participants could not predict the ear of target presentation. Thus, we expected that both left and right target presentations in the uninformative condition would be preceded by a similar pattern of alpha oscillations, during the cue-target delay. Behaviorally, we have assessed the influence of the cue category (2 levels: informative and uninformative) and target side presentation (left and right) on median reaction times (see figure below). As expected, we found only a significant cue category effect (p &lt; 0.001) and a significant target side presentation effect (p = 0.015) with no significant interaction (p = 0.18) (see Figure A). Shorter reaction times to right than to left targets, irrespective of the cue type, is consistent with the well-known right ear advantage and is not likely to have biased our results during the delay preceding target sound presentation.

I think that the paper could be improved by organizing data analysis section better. The different sections seem somewhat disconnected from each other. Maybe having a sentence at the start of each subsection explaining the purpose of each analysis described would help.

We agree with the reviewer's concerns about the materials and methods section. We have indeed incorporated an outline (section 2.4.2.) of the data analysis to help readers navigate through this section. In an attempt, to make it clearer, we have added sentences to stress out the purpose of each analysis.

Under section 2.6.4 it is stated that alpha power was analyzed in the two frequency bands 7-11 Hz and 11-15Hz. Why did authors choose to overlap the two bands?

The frequency resolution for DICS analysis depends on the Rayleigh frequency of the data which is equal to 1/t, with t being the duration (in seconds) of the period of interest. Since we were interested in reconstructing activity of 0.4 sec windows (plus 25ms at the edges of each window), we were restricted to a frequency resolution of approximately 2.2Hz. Therefore, we were obliged to adapt the frequency range according to the frequency resolution to 9Hz ± 2Hz (7-11 Hz) and 13Hz ± 2Hz (11-15 Hz).

However, it is important to note that our analysis went past this limitation by reconstructing the time-course of source activity (virtual electrodes). This method involves multiplying sensor-level data by LCMV beamformer spatial filters. The output is at the same sampling frequency (600 Hz) than the original sensor level data. Thus, by applying Morlet Wavelets to virtual-electrode data with a large time window (-3 to 3 seconds, relative to cue onset), we could achieve a superior frequency resolution allowing to investigate the alpha sub-bands with less overlap.

It would be helpful to the reader if Table 1 indicated increases and decreases at different the frequency bands.

We have indeed added up and down arrows to Table1 to indicate whether source-level observed effects were an increase or decrease in alpha power, respectively.

In Figure 6 alpha was averaged in different time window 900-1200 ms and from 10-12 Hz. It was not completely clear from the text why time window and frequency range changed?

For analysis at the source level, we chose the time-window showing a significant effect at the sensor level (900-1200 ms). Similar to the previously raised concern about the frequency overlap, the frequency resolution was constrained by the duration of this time-window to 3.3 Hz. Therefore, we were obliged to adapt the frequency range according to the frequency resolution to 13Hz ± 3Hz (10-16 Hz).

I wonder if authors could use a better brain model in Figure 6C to overlay the results of source analysis.

We understand the reviewer's concerns. However, to the best of our knowledge and the options provided in the fieldtrip toolbox, this source model provides the better spatial resolution to demonstrate the distribution of the correlation coefficients.

The correlational analyses are interesting. However, I could not tell how/whether they were corrected for the multiple comparison.

The correlational analyses have been indeed corrected for multiple corrections. As stated in the Materials and Methods section 2.6.6: “Topographies were subsequently subjected to a one-sample t-test of the correlations and nonparametric cluster-based permutation analysis to correct for multiple comparisons in the sensor space, time and frequency dimensions.”

Also, in section 2.6.7: “The statistical significance of the correlations was assessed at the group level with a one-sample t-test of the correlation z-values at each grid point and then subjected to a cluster-level randomization test to correct for multiple comparisons in the source space dimension.”

Reviewer #2: This is a carefully collected study providing a nice replication of a reported finding on alpha power fluctuations regulating activity beyond the visual modality. As such strengthening the notion of alpha activity organizing functional brain architecture. The reported findings are of relevance for a broad readership and will strengthen understanding and further theoretical development in the field of cognitive neuroscience. However, several points need to be addressed:

“Only this power increase in high alpha was found correlated with behavior”. This specificity claim required test of the homogeneity of regression slopes. This test is lacking yet crucial.

We are not sure to understand the reason why we should test for the homogeneity of regression slopes since we do not perform a multiple regression analysis nor an ANCOVA. Indeed, we used a non-parametric approach used by Mazaheri and colleagues (2013), which rely on multiple spearman tests of correlation at each time-frequency point followed by a correction for multiple tests using non-parametric cluster-based permutations. The steps of this approach are detailed below.

1. For each participant, we performed non-parametric Spearman tests of correlation across trials between RT and alpha power at each sensor for each time-frequency point (between 6 and 16Hz, by step of 1 Hz, and between 0 and 1200 ms post-cue onset, by step of 50 ms), separately.

2. The obtained correlation coefficients were then converted to z-values using Fisher's r- to z-transformation to obtain normally distributed variables.

3. Finally, the statistical significance of the correlations was assessed at the group level, by comparing to 0, the obtained z-values at each sensor, for each time-frequency point, using a one-sample t-test across participants.

4. Non-parametric cluster-based permutations across participants were used to correct for multiple tests in the sensor, time and frequency dimensions. In brief (as explained in section (2.6.1), we first calculate one-sample t-tests for each sensor at each time-frequency point, which are then thresholded at P &lt; 0.05. The sum within each cluster (Tsum) is retained, and the procedure is repeated 1000 times on shuffled data. On each permutation, the maximum Tsum (Tmax) is retained yielding a distribution of 1000 Tmax values. From this distribution, the cluster probability of each empirically observed Tsum can be derived. Clusters are labelled as significant with a P-value = 0.05.

The corresponding paragraphs in the Method sections have been modified to clarify this statistical approach.

Moreover, we modified the sentence cited by the reviewer: “Only this power increase in high alpha was significantly correlated with behavior”, as well as other similar sentences throughout the manuscript.

Saccades are not controlled. It is now well established that saccades can trigger and affect alpha power fluctuations substantially. Please calculate and provide an illustration of saccade direction as a function of left, right, and uninformative cue. This can be done either on the EOG or frontal SQUIDS data.

We understand the reviewer's concern about saccade artefact as confounding variable in electrophysiological data. However, the impact of this artefact is most pronounced in the gamma-range (Carl et al., 2013). Nonetheless, using the EOG, in each condition, we have detected the saccades and their direction by means of thresholding (threshold = 4) the z-transformed values of the EOG data. We found a low % of trials contaminated by a saccade (around 2%). Next, we applied a linear mixed-effects model to investigate the effects of cue condition (3 levels: left, right and uninformative) and saccade direction (2 levels: towards the right and towards the left) on the percentage of saccade-contaminated trials. We have found only a significant main effect of saccade direction (p &lt; 0.001), irrespective of the cue condition (see Figure B). This strongly suggests that effects reported in the manuscript are not likely to be explained by differences in saccade proportions between cue conditions.

Which hand was used for joystick control? It is well established that execution and/or preparation for motor output is associated with contra lateral decrease in high alpha / beta power in parallel to ipsi lateral increase. This is somewhat the pattern of activity illustrated in Figure 6A for example.

All participants were right handed. Using their right index, participants either pushed or pulled a joystick to indicate the pitch of the target sound. We agree with the reviewer that the topography of power previously shown on figure 6a resembles the topography for preparation of a right motor response. However, the correlation between power and reaction times is mostly significant posteriorly to the central gyrus in low parietal and occipital regions, according to analysis at the source level (Fig 6c). Therefore, correlation results are not likely to be related to motor preparation processing. Please note that Figure 6a has been change in response to another comment of the same reviewer (see in the following).

Averaging of time courses within ROIs is dangerous due to different (arbitrary) polarities of beamforming results. I suggest TFR prior to averaging across dipoles within ROIs. Does this affect Figure 5?

The choice to average time-courses within each ROI before computing TFR was computation-efficiency related. As suggested by the reviewer, we re-analyzed the data by computing TFR before averaging across dipoles. As seen in the figure C below, while the power estimate changes between the two methods, the effects between conditions remain highly comparable.

Also, size asymmetry between left &lt; right Heschl's gyrus would likely bias power estimates. Yet, these are the basis for the right AC claim. How many dipoles were included per auditory parcel? Was that done for Heschl Gyrus or also Planum temporale included?

We have defined 4 auditory ROIs, one for Heschl's gyrus and one in the posterior STG (including the Planum Temporale) in each hemisphere. Within each ROI, we picked up for further analysis 5 neighboring dipoles exhibiting the strongest alpha power modulations in contrast to baseline. Hence, we doubt that there would be any bias power estimates since the same number of dipoles (voxels) were considered in all parcels.

What was the regularization parameter for DICS? Please re-compute with smallest value possible or even without any regularization on the raw non-ICAed data. Are there “real” activations of AC or a “leakage” of posterior activation due to regularization?

We used a regularization parameter of 5% for DICS analysis. We have opted for the somewhat standard regularization value (5%) that has been used previously in the auditory alpha literature (e.g. Weise et al., 2016). We have re-computed the DICS analysis with smaller regularization values (1 and 0.1 %) from ICAed data and with a 5% regularization from non-ICAed data. The results are presented in the figure D below for the two time-windows of interest (200-600ms and 600-1000ms) and two frequency bands (low and high alpha).

As shown in the figure D, source estimations are rather similar regardless the regularization or the application of ICA to the data. Therefore, using a 5% regularization value and ICAed data for eye-related artefacts seem to be an appropriate approach which provides stable source reconstruction with minimal spatial leakage (Brookes et al., 2008). These results rather speak against a leakage of posterior activation towards the auditory cortices.

Correlation between alpha and reaction time is computed using sliding window of 100 ms. This gives a frequency resolution of 0 10 20 30 etc. Hz. Yet 7 and 15 are not on that list. Please re-calculate this analysis using fft and not sliding window approach over the entire 1.2 sec period. This gives the desired freq resolution in steps of 1 Hz and allows for reliably estimated power values.

We have not used the aforementioned sliding window to compute an FFT on the time signal at the sensor level. We have actually calculated a time-frequency (TF) transform of the time signal using gaussian Morlet's wavelets. It was then possible to arbitrarily sample the T-F domain. Due to the gaussian shape of the Morlet's wavelet, each TF point corresponds to a TF resolution of 2.st x 2.sf (see Tallon-Baudry & Bertrand, TICS, 1999). Since we have used Morlet's wavelet defined by a constant value m = f/sf =7, we had a varying resolution in the TF domain ranging from +/- 160ms x +/- 1Hz at 7Hz, to +/- 80ms x +/- 2Hz at 14 Hz.

We have recomputed the test by calculating correlation coefficients between alpha activity and reaction times at each sensor and at each frequency (between 6--16Hz) and time (0--1200 ms post-cue onset) point with no averaging or smoothing using the original TF data (by steps of 50ms and 1 Hz). We used Cluster Based Permutation testing to correct for multiple tests and to identify the time-frequency region showing a significant correlation at the sensor level. Figure 6 has been modified accordingly. In addition, the material and methods section has been updated.

Correlations with behavior in figure 6 flip polarity from sensor to source space- why?

We believe that figure 6 was misleading since on the sensor level we reported power-values while in the source level we reported t-values of the correlation significance test. Therefore, we have changed the figure to avoid any future confusion and plot the t-values of the correlation significance test at both the sensor and source levels.

Please explain what “Partial Cannonical Correlation (PCC) beamformer” is and how it works. Also why using PCC and not LCMV or DICS filters multiplied with single trial sensor data?

The Partial Cannonical Correlation (PCC) beamformer is very similar to the DICS beamformer. However, it takes significantly less time to estimate source activity at a single-trial level. Thus, due to the substantial number of voxels (47520) for each trial for each participant, the PCC provided a much more computationally efficient alternative to the DICS. For more details, please consult https://goo.gl/J8hBDU.

## References

[B1] Aguera PE, Jerbi K, Caclin A, Bertrand O (2011) ELAN: a software package for analysis and visualization of MEG, EEG, and LFP signals. Comput Intell Neurosci 2011:158970 10.1155/2011/158970 21687568 PMC3113286

[B2] Başar E (2012) A review of alpha activity in integrative brain function: fundamental physiology, sensory coding, cognition and pathology. Int J Psychophysiol 86:1–24. 10.1016/j.ijpsycho.2012.07.002 22820267

[B3] Bates D, Mächler M, Bolker B, Walker S (2014) Fitting linear mixed-effects models using lme4. arXiv Prepr arXiv14065823.

[B4] Berger H (1929) Über das elektrenkephalogramm des menschen. Eur Arch Psychiatry Clin Neurosci 87:527–570. 10.1007/BF01797193

[B5] Bidet-Caulet A, Fischer C, Besle J, Aguera P-E, Giard M-H, Bertrand O (2007) Effects of selective attention on the electrophysiological representation of concurrent sounds in the human auditory cortex. J Neurosci 27:9252–9261. 10.1523/JNEUROSCI.1402-07.2007 17728439 PMC6673135

[B6] Bidet-Caulet A, Mikyska C, Knight RT (2010) Load effects in auditory selective attention: evidence for distinct facilitation and inhibition mechanisms. Neuroimage 50:277–284. 10.1016/j.neuroimage.2009.12.039 20026231 PMC2819629

[B7] Bidet-Caulet A, Buchanan KG, Viswanath H, Black J, Scabini D, Bonnet-Brilhault F, Knight RT (2015) Impaired facilitatory mechanisms of auditory attention after damage of the lateral prefrontal cortex. Cereb Cortex 25:4126–4134. 10.1093/cercor/bhu131 24925773 PMC4626830

[B8] Chait M, de Cheveigné A, Poeppel D, Simon JZ (2010) Neural dynamics of attending and ignoring in human auditory cortex. Neuropsychologia 48:3262–3271. 10.1016/j.neuropsychologia.2010.07.007 20633569 PMC2926275

[B67] Cohen J (1988) Statistical power analysis for the behavioral sciences. 2nd.

[B9] Corbetta M, Shulman GL (2002) Control of goal-directed and stimulus-driven attention in the brain. Nat Rev Neurosci 3 10.1038/nrn755 11994752

[B10] de Fockert JW, Lavie N (2001) The role of working memory in visual selective attention. Sci 291:1803–1806. 10.1126/science.1056496 11230699

[B11] Faul F, Erdfelder E, Lang AG, Buchner A (2007) G*Power 3: a flexible statistical power analysis program for the social, behavioral, and biomedical sciences. Behav Res Methods 39:175–191. 10.3758/BF03193146 17695343

[B12] Faul F, Erdfelder E, Buchner A, Lang AG (2009) Statistical power analyses using G* Power 3.1: tests for correlation and regression analyses. Behav Res Methods 41:1149–1160. 10.3758/BRM.41.4.1149 19897823

[B13] Foxe JJ, Snyder AC (2011) The role of alpha-band brain oscillations as a sensory suppression mechanism during selective attention. Front Psychol 2:154 10.3389/fpsyg.2011.00154 21779269 PMC3132683

[B14] Foxe JJ, Simpson GV, Ahlfors SP (1998) Parieto‐occipital ∼10 Hz activity reflects anticipatory state of visual attention mechanisms. Neuroreport 9:3929–3933. 10.1097/00001756-199812010-00030 9875731

[B15] Frey JN, Mainy N, Lachaux J-P, Muller N, Bertrand O, Weisz N (2014) Selective modulation of auditory cortical alpha activity in an audiovisual spatial attention task. J Neurosci 3:6634–6639. 24806688 10.1523/JNEUROSCI.4813-13.2014 PMC6608137

[B16] Frey JN, Ruhnau P, Weisz N (2015) Not so different after all: the same oscillatory processes support different types of attention. Brain Res 1626:183–197. 10.1016/j.brainres.2015.02.017 25721788

[B17] Fu KMG, Foxe JJ, Murray MM, Higgins BA, Javitt DC, Schroeder CE (2001) Attention-dependent suppression of distracter visual input can be cross-modally cued as indexed by anticipatory parieto-occipital alpha-band oscillations. Cogn Brain Res 12:145–152. 10.1016/S0926-6410(01)00034-9 11489617

[B18] Gazzaley A, Cooney JW, McEvoy K, Knight RT, D’Esposito M (2005) Top-down enhancement and suppression of the magnitude and speed of neural activity. J Cogn Neurosci 17:507–517. 10.1162/0898929053279522 15814009

[B19] Gazzaley A, Clapp W, Kelley J, McEvoy K, Knight RT, D’Esposito M (2008) Age-related top-down suppression deficit in the early stages of cortical visual memory processing. Proc Natl Acad Sci USA 105:13122–13126. 10.1073/pnas.0806074105 18765818 PMC2529045

[B20] Golob EJ, Pratt H, Starr A (2002) Preparatory slow potentials and event-related potentials in an auditory cued attention task. Clin Neurophysiol 113:1544–1557. 12350430 10.1016/s1388-2457(02)00220-1

[B21] Gomez-Ramirez M, Kelly SP, Molholm S, Sehatpour P, Schwartz TH, Foxe JJ (2011) Oscillatory sensory selection mechanisms during intersensory attention to rhythmic auditory and visual inputs: a human electrocorticographic investigation. J Neurosci 31:18556–18567. 10.1523/JNEUROSCI.2164-11.2011 22171054 PMC3298747

[B22] Groppe DM, Bickel S, Keller CJ, Jain SK, Hwang ST, Harden C, Mehta AD (2013) Dominant frequencies of resting human brain activity as measured by the electrocorticogram. Neuroimage 79:223–233. 10.1016/j.neuroimage.2013.04.044 23639261 PMC4269223

[B23] Gross J, Kujala J, Hamalainen M, Timmermann L, Schnitzler A, Salmelin R (2001) Dynamic imaging of coherent sources: studying neural interactions in the human brain. Proc Natl Acad Sci USA 98:694–699. 10.1073/pnas.98.2.694 11209067 PMC14650

[B24] Haegens S, Luther L, Jensen O (2012) Somatosensory anticipatory alpha activity increases to suppress distracting input. J Cogn Neurosci 24:677–685. 10.1162/jocn_a_00164 22066587

[B25] Haegens S, Cousijn H, Wallis G, Harrison PJ, Nobre AC (2014) Inter- and intra-individual variability in alpha peak frequency. Neuroimage 92:46–55. 10.1016/j.neuroimage.2014.01.049 24508648 PMC4013551

[B26] Haegens S, Barczak A, Musacchia G, Lipton ML, Mehta AD, Lakatos P, Schroeder CE (2015) Laminar profile and physiology of the α rhythm in primary visual, auditory, and somatosensory regions of neocortex. J Neurosci 35:14341–14352. 10.1523/JNEUROSCI.0600-15.2015 26490871 PMC4683691

[B27] Hillyard SA, Vogel EK, Luck SJ (1998) Sensory gain control (amplification) as a mechanism of selective attention: electrophysiological and neuroimaging evidence. Philos Trans R Soc London B Biol Sci 353:1257–1270. 10.1098/rstb.1998.0281 9770220 PMC1692341

[B28] Hyde KL, Peretz I, Zatorre RJ (2008) Evidence for the role of the right auditory cortex in fine pitch resolution. Neuropsychologia 46:632–639. 10.1016/j.neuropsychologia.2007.09.004 17959204

[B29] Jensen O, Mazaheri A (2010) Shaping functional architecture by oscillatory alpha activity: gating by inhibition. Front Hum Neurosci 4:186 10.3389/fnhum.2010.00186 21119777 PMC2990626

[B30] Jiang H, Bahramisharif A, van Gerven MAJ, Jensen O (2015) Measuring directionality between neuronal oscillations of different frequencies. Neuroimage 118:359–367. 10.1016/j.neuroimage.2015.05.044 26025291

[B31] Kawasaki M, Kitajo K, Yamaguchi Y (2010) Dynamic links between theta executive functions and alpha storage buffers in auditory and visual working memory. Eur J Neurosci 31:1683–1689. 10.1111/j.1460-9568.2010.07217.x 20525081 PMC2878597

[B32] Kelly SP, Lalor EC, Reilly RB, Foxe JJ (2006) Increases in alpha oscillatory power reflect an active retinotopic mechanism for distracter suppression during sustained visuospatial attention. J Neurophysiol 95:3844–3851. 10.1152/jn.01234.2005 16571739

[B33] Klimesch W (1999) EEG alpha and theta oscillations reflect cognitive and memory performance: a review and analysis. Brain Res Brain Res Rev 29:169–195. 10209231 10.1016/s0165-0173(98)00056-3

[B34] Klimesch W (2012) Alpha-band oscillations, attention, and controlled access to stored information. Cell 16:606–617. 10.1016/j.tics.2012.10.007 PMC3507158 23141428

[B35] Klimesch W, Schimke H, Pfurtscheller G (1993) Alpha frequency, cognitive load and memory performance. Brain Topogr 5:241–251. 8507550 10.1007/BF01128991

[B36] Klimesch W, Sauseng P, Hanslmayr S (2007) EEG alpha oscillations: the inhibition–timing hypothesis. Brain Res Rev 53:63–88. 10.1016/j.brainresrev.2006.06.003 16887192

[B37] Lattner S, Meyer ME, Friederici AD (2005) Voice perception: sex, pitch, and the right hemisphere. Hum Brain Mapp 24:11–20. 10.1002/hbm.20065 15593269 PMC6871712

[B38] Maris E, Oostenveld R (2007) Nonparametric statistical testing of EEG- and MEG-data. J Neurosci Methods 164:177–190. 10.1016/j.jneumeth.2007.03.024 17517438

[B39] Mazaheri A, van Schouwenburg MR, Dimitrijevic A, Denys D, Cools R, Jensen O (2013) Region-specific modulations in oscillatory alpha activity serve to facilitate processing in the visual and auditory modalities. Neuroimage 87:356–362. 10.1016/j.neuroimage.2013.10.052 24188814

[B40] Milner B (1962) Laterality effects in audition In: Interhemispheric relations and cerebral dominance. Baltimore, MD: Johns Hopkins University Press.

[B41] Müller N, Weisz N (2012) Lateralized auditory cortical alpha band activity and interregional connectivity pattern reflect anticipation of target sounds. Cereb Cortex 22:1604–1613. 10.1093/cercor/bhr232 21893682

[B42] Nobre AC, Sebestyen GN, Gitelman DR, Mesulam MM, Frackowiak RS, Frith CD (1997) Functional localization of the system for visuospatial attention using positron emission tomography. Brain J Neurol 120:515–533. 10.1093/brain/120.3.515 9126062

[B43] Nolte G (2003) The magnetic lead field theorem in the quasi-static approximation and its use for magnetoencephalography forward calculation in realistic volume conductors. Phys Med Biol 48:3637. 14680264 10.1088/0031-9155/48/22/002

[B44] Oostenveld R, Fries P, Maris E, Schoffelen JM (2011) FieldTrip: open source software for advanced analysis of MEG, EEG, and invasive electrophysiological data. Comput Intell Neurosci 2011:156869 10.1155/2011/156869 21253357 PMC3021840

[B45] Pfurtscheller G, Stancák A Jr, Neuper C (1996) Event-related synchronization (ERS) in the alpha band—an electrophysiological correlate of cortical idling: a review. Int J Psychophysiol 24:39–46. 8978434 10.1016/s0167-8760(96)00066-9

[B46] Requin J, Brener J, Ring C (1991) Preparation for action In: Handbook of cognitive psychophysiology: central and autonomic nervous system approaches. New York: Wiley & Sons.

[B47] Rihs TA, Michel CM, Thut G (2007) Mechanisms of selective inhibition in visual spatial attention are indexed by alpha-band EEG synchronization. Eur J Neurosci 25:603–610. 10.1111/j.1460-9568.2007.05278.x 17284203

[B48] Rihs TA, Michel CM, Thut G (2009) A bias for posterior alpha-band power suppression versus enhancement during shifting versus maintenance of spatial attention. Neuroimage 44:190–199. 10.1016/j.neuroimage.2008.08.022 18793732

[B49] Sauseng P, Klimesch W, Stadler W, Schabus M, Doppelmayr M, Hanslmayr S, Gruber WR, Birbaumer N (2005) A shift of visual spatial attention is selectively associated with human EEG alpha activity. Eur J Neurosci 22:2917–2926. 10.1111/j.1460-9568.2005.04482.x 16324126

[B50] Searle SR, Speed FM, Milliken GA (1980) Population marginal means in the linear model: an alternative to least squares means. Am Stat 34:216–221. 10.2307/2684063

[B51] Slagter HA, Prinssen S, Reteig LC, Mazaheri A (2016) Facilitation and inhibition in attention: functional dissociation of pre-stimulus alpha activity, P1, and N1 components. Neuroimage 125:25–35. 10.1016/j.neuroimage.2015.09.058 26436713

[B52] Spierer L, Bellmann-Thiran A, Maeder P, Murray MM, Clarke S (2009) Hemispheric competence for auditory spatial representation. Brain 132:1953–1966. 10.1093/brain/awp127 19477962

[B53] Spierer L, De Lucia M, Bernasconi F, Grivel J, Bourquin NMP, Clarke S, Murray MM (2011) Learning-induced plasticity in human audition: objects, time, and space. Hear Res 271:88–102. 10.1016/j.heares.2010.03.086 20430070

[B54] Tallon-Baudry C, Bertrand O (1999) Oscillatory gamma activity in humans and its role in object representation. Trends Cogn Sci 3:151–162. 10322469 10.1016/s1364-6613(99)01299-1

[B55] R Team (2014) R: a language and environment for statistical computing. Vienna: R Foundation for Statistical Computing.

[B56] Thut G (2006) alpha-Band electroencephalographic activity over occipital cortex indexes visuospatial attention bias and predicts visual target detection. J Neurosci 26:9494–9502. 10.1523/JNEUROSCI.0875-06.2006 16971533 PMC6674607

[B57] Tzourio-Mazoyer N, Landeau B, Papathanassiou D, Crivello F, Etard O, Delcroix N, Mazoyer B, Joliot M (2002) Automated anatomical labeling of activations in SPM using a macroscopic anatomical parcellation of the MNI MRI single-subject brain. Neuroimage 15:273–289. 10.1006/nimg.2001.0978 11771995

[B58] van Diepen RM, Mazaheri A (2017) Cross-sensory modulation of alpha oscillatory activity: suppression, idling, and default resource allocation. Eur J Neurosci 45:1431–1438. 10.1111/ejn.13570 28378515

[B59] Van Veen BD, van Drongelen W, Yuchtman M, Suzuki A (1997) Localization of brain electrical activity via linearly constrained minimum variance spatial filtering. Biomed Eng IEEE Trans 44:867–880. 10.1109/10.623056 9282479

[B60] Von Békésy G, Wever EG (1960) Experiments in hearing. New York: McGraw-Hill.

[B61] Weise A, Hartmann T, Schröger E, Weisz N, Ruhnau P (2016) Cross-modal distractors modulate oscillatory alpha power: the neural basis of impaired task performance. Psychophysiology 53:1651–1659. 10.1111/psyp.12733 27468982

[B62] Weisz N, Hartmann T, Müller N, Lorenz I, Obleser J (2011) Alpha rhythms in audition: cognitive and clinical perspectives. Front Psychol 2:73 10.3389/fpsyg.2011.00073 21687444 PMC3110491

[B63] Weisz N, Muller N, Jatzev S, Bertrand O (2014) Oscillatory alpha modulations in right auditory regions reflect the validity of acoustic cues in an auditory spatial attention task. Cereb Cortex 24:2579–2590. 10.1093/cercor/bht113 23645711

[B64] Zatorre RJ, Belin P (2001) Spectral and temporal processing in human auditory cortex. Cereb Cortex 11:946–953. 11549617 10.1093/cercor/11.10.946

[B65] Zatorre RJ, Penhune VB (2001) Spatial localization after excision of human auditory cortex. J Neurosci 21:6321–6328. 11487655 10.1523/JNEUROSCI.21-16-06321.2001 PMC6763137

[B66] Zatorre RJ, Belin P, Penhune VB (2002) Structure and function of auditory cortex: music and speech. Trends Cogn Sci 6:37–46. 11849614 10.1016/s1364-6613(00)01816-7

